# Influence of the dental topical application of a nisin-biogel in the oral microbiome of dogs: a pilot study

**DOI:** 10.7717/peerj.11626

**Published:** 2021-07-14

**Authors:** Eva Cunha, Sara Valente, Mariana Nascimento, Marcelo Pereira, Luís Tavares, Ricardo Dias, Manuela Oliveira

**Affiliations:** 1 CIISA - Centro de Investigação Interdisciplinar em Sanidade Animal, Faculdade de Medicina Veterinária, Universidade de Lisboa, Lisboa, Portugal; 2 BioISI: Biosystems & Integrative Sciences Institute, Faculdade de Ciências, Universidade de Lisboa, Lisboa, Portugal

**Keywords:** Nisin-biogel, Oral microbiome, Periodontal disease, Dogs

## Abstract

Periodontal disease (PD) is one of the most widespread inflammatory diseases in dogs. This disease is initiated by a polymicrobial biofilm in the teeth surface (dental plaque), leading to a local inflammatory response, with gingivitis and/or several degrees of periodontitis. For instance, the prevention of bacterial dental plaque formation and its removal are essential steps in PD control. Recent research revealed that the antimicrobial peptide nisin incorporated in the delivery system guar gum (biogel) can inhibit and eradicate bacteria from canine dental plaque, being a promising compound for prevention of PD onset in dogs. However, no information is available regarding its effect on the dog’s oral microbiome. In this pilot study, the influence of the nisin-biogel on the diversity of canine oral microbiome was evaluated using next generation sequencing (NGS), aiming to access the viability of nisin-biogel to be used in long-term experiment in dogs. Composite toothbrushing samples of the supragingival plaque from two dogs were collected at three timepoints: T1—before any application of the nisin-biogel to the animals’ teeth surface; T2—one hour after one application of the nisin-biogel; and T3—one hour after a total of three applications of the nisin-biogel, each 48 hours. After that, microbial profiling was performed by NGS of the V3V4 16s rRNA region. After only one application of the nisin-biogel to the oral cavity of dogs, a statistically significant reduction in microbial diversity was observed (T2) as well as a reduction of some bacterial species potentially related with distinct stages of PD, when compared with samples collected before any application (T1). However, after a total of three nisin-biogel applications (T3), a recovery of the microbial diversity was detected. In conclusion, the nisin-biogel may influence the canine oral microbiome. A reduction in some bacterial species potentially related with distinct stages of PD was observed. This pilot study will help to design a controlled *in vivo* clinical trial to evaluate nisin-biogel effect on dental plaque progression and canine periodontal indices evolution in a long-term application period.

## Introduction

The canine oral cavity is a complex and highly diverse environment. Within this ecosystem, the oral microbial community can change depending on several factors, including the status of the animal’s oral health (Ruparell et al., 2020; Wu et al., 2020). Evaluation of the microbiome is a powerful weapon for the study of oral health, oral diseases or even the effect of oral treatments. Nowadays, genetics and bioinformatics innovative methodologies, such as high-throughput analysis through next generation sequencing (NGS), allow to evaluate the true diversity of *in vivo* bacterial communities (Teles et al., 2013; Wu et al., 2020; Wallis et al., 2021). Using these methodologies, Riggio et al. (2011) have been able to characterize the canine oral microbiome in heathy dogs or in animals with periodontal disease (PD) including animals with gingivitis and periodontitis; and Wallis et al. (2015) evaluated changes in subgingival bacterial communities during the transition from mild gingivitis to early stages of periodontitis. In addition, Dewhirst et al. (2012) evaluated the subgingival plaque and showed that the healthy oral microbiome of dogs is mainly composed by Phyla Firmicutes (45.9%), Proteobacteria (14.7%), Bacteroidetes (12.2%), Spirochetes (10.5%), Synergistes (3.7%), Actinobacteria (3.4%) and Fusobacteria (2.8%). Similar results were obtained by Davis et al. (2013) with subgingival plaque samples, and by Holcombe et al. (2014) and Ruparell et al. (2020) using supragingival plaque samples. At genus level *Pasteurella*, *Bergeyella*, *Conchiformibius*, *Porphyromonas*, *Actinomyces*, *Fusobacterium* and *Neisseria* were described as predominant in healthy canine oral samples (Oh et al., 2015; Ruparell et al., 2020). However, many differences can be observed related to individual differences, environmental and alimentary conditions, or even disease establishment and progression. For example, reports describe *Porphyromonas cangingivalis* as the most abundant species in the oral cavity of dogs with or without gingivitis, other species like *Neisseria* spp. and *Bergeyella* spp., described as early colonizers, are usually present in the dental plaque of healthy animals, but may also be present in dogs with gingivitis (PD stage 1), on the other hand *P. gulae* or *Peptostreptococcus* spp. are often linked to mild periodontitis (PD stage 2) and possibly PD stage 3 or 4 (Lawson et al., 2012; Davis et al., 2013; Holcombe et al., 2014; Polkowska, Sobczyńska-Rak & Gołyńska, 2014; Kačírová et al., 2019; Ruparell et al., 2020).

Recently, a promising antimicrobial compound composed by nisin incorporated in the delivery system guar gum (biogel), has been proposed as an alternative measure for the control of PD in dogs (Cunha et al., 2018; Cunha et al., 2020). PD is a common inflammatory disease in dogs, caused by a polymicrobial biofilm in the teeth surface and a subsequent local inflammatory response, that leads to gingivitis and/or periodontitis (Niemiec, 2008; Teles et al., 2013; Stepaniuk, 2019). Nisin is a bacteriocin produced mainly by *Lactococcus lactis*, which presents antimicrobial activity against several periodontal pathogens (Shin et al., 2016; Cunha et al., 2018; Naguyen et al., 2020). Acting by linkage to the bacterial membrane lipid II, nisin promotes inhibition of cell wall synthesis and membrane disruption with pore formation (Shin et al., 2016). In addition, *in vivo* studies have showed that nisin has the capacity to reduce dental plaque and gingivitis (Howell et al., 1993; Shin et al., 2016; Naguyen et al., 2020). In previous studies, the nisin-biogel showed ability to inhibit and eradicate canine PD-related biofilms (formed by bacteria collected from canine dental plaque), maintained its activity in the presence of canine saliva, and showed no toxicity to eukaryotic cells in concentrations up to 200 µg/mL (Shin et al., 2015; Cunha et al., 2018; Cunha et al., 2020). Despite that, no information regarding nisin’s influence on the canine oral microbiome is available. Considering that, a pilot study was performed, representing a preliminary small-scale study aiming to investigate if a randomized controlled main clinical trial would be feasible.

In this work we aimed to evaluate the influence of the nisin-biogel on the oral microbiome of healthy dogs, after one topical application of nisin-biogel in the teeth surface (T2) and after a total of three applications, with 48 hours interval each (T3), in comparison with the commensal canine oral microbiome, before any application (T1), using supragingival composite samples.

## Materials & methods

### Animals’ selection

Two dogs were selected to participate in this experiment, after evaluation by a veterinary surgeon, which included periodontal evaluation according to the American Veterinary Dental College guidelines. Inclusion criteria included: healthy animals, with more than 2 years, without periodontal disease or any other disease and no history of antimicrobial therapy in the last 6 months. To guarantee that no bias resulted from applied procedures, dogs were also selected based on their acceptance towards biogel topical application and sample collection.

The inclusion of these dogs was performed after owner’s written consent and approval by the Ethical Committee for Research and Teaching (CEIE) of the Faculty of Veterinary Medicine—University of Lisbon, Portugal (N/Refª 014/2020).

### Nisin-biogel preparation

Nisin stock solution (1,000 µg/mL) was prepared by dissolving 1 g of nisin powder (2.5% purity, 1,000 IU/mg, Sigma–Aldrich, St. Louis, MO, USA) in 25 mL of HCL (0.02M) (Merk, Darmstadt, Germany). Stock solution was filtered using a 0.22 µm cellulose acetate filter. Biogel at 1.5% (w/v) was prepared by dissolving 0.75 g of guar gum (Sigma–Aldrich, St. Louis, MO, USA) in 50 mL of sterile distilled water followed by heat sterilization by autoclave. Then, nisin solution was incorporated within the biogel, in a proportion of 1:1, to achieve a final nisin concentration of 200 µg/mL (Cunha et al., 2018).

### Nisin-biogel application and oral sampling

Nisin-biogel (200 µg/mL) was topically applied to all the gingival margin and dental surface of both animals by a veterinary surgeon, 2 h after meals. A total of two mL of nisin-biogel was applied, at 48 h intervals, in a total of three applications per dog (1 week). Composite supragingival plaque samples were collected from the teeth surface (maxillary and mandibular teeth) of all teeth, with a sterile toothbrush. Sample collection was performed on three timepoints: T1—before any nisin-biogel application; T2—one hour after the first nisin-biogel application; T3—one hour after the last nisin-biogel application. Two samples were collected, from each animal, in all timepoints.

### DNA extraction

A modified version of the guanidium thiocyanate protocol, described by Pitcher, Saunders & Owen (1989), was used for DNA extraction (Pitcher, Saunders & Owen, 1989). After, samples were purified with SeraMag Carboxylate Modified beads^®^ (GE Healthcare Life Sciences, Marlborough, MA, USA) in a binding buffer (10 mM Tris base, 1 mM EDTA, 2.5 M NaCl, 20% PEG 8000, 0.05% Tween 20, pH 8.0), washed in 70% ethanol and eluted in TE buffer. Quality control was performed by Qubit^®^ quantification and Nanodrop^®^ absorbance determination.

### Amplification of 16S rRNA

For amplification of bacterial 16S rRNA genes of the total bacterial community, an universal primer set, 341F (5′- CCTACGGGAGGCAGCAG-3′) and 907R (5′- CCGTCAATTCMTTTGAGTTT-3′) was used. The PCR mixture (25 µL) was composed by 12.5 µL of LongAmp^®^ 2x Taq Master Mix (NEB, USA), 1 µL of purified DNA, 2 µL of each primer (5 pM) and 7.5 µl of nuclease free water (ThermoFisher Scientific, Waltham, MA, USA). PCR cycling conditions consisted of an initial denaturation step at 94 °C (3 min), followed by 35 cycles of 94 °C (20 s), 45 °C (30 s) and 65 °C (60 s) and a final extension at 65 °C (10 min). Successful amplification was confirmed through electrophoresis (90 V, 60 min) of the PCR products on 1.2% agarose gel.

### Sequencing and data processing

Microbial profiling was performed by NGS of the V3V4 16S rRNA region. Each sample and sequencing run were carried out on MySeq Illumina sequencing platform. The resulting sequencing data was analyzed by Qiime2 pipeline for taxonomic classification of 16S sequencing metagenomic data. Quality control was performed with Debur workflow with trimming of 150 bps. Phred quality score of 20 was used for assessment of sequence quality, allowing a base accuracy of 99%. Phylogenetic diversity analysis was performed with phylogeny align-to-tree-fast-tree. A multiple sequence alignment of the sequences was performed, removing positions that are highly variable. Samples reads were subsampled to 90,337 reads per sample, which correspond to the sample with the smallest number of reads. Rarefaction analysis was considered for alpha diversity as a function of sampling depth, established at the median depth between all samples (134,632 reads). Database Greengenes99 version 13.8 (203,435 OTUs) was used for taxonomic classification. Diversity between timepoints was evaluated by Shannon index, which accounts for both richness and evenness of the present species.

### Statistical analysis

Diversity differences between timepoints were evaluated by factor analysis Kruskall–Wallis sum rank test. A confidence interval of 95% was considered, with a *p*-value ≤ 0.05 indicating statistical significance. Quantitative variables are expressed as mean values ± standard deviation.

## Results

### Animal’s selection

Two male dogs were included in the study, with no history of previous diseases or antimicrobial treatments in the past 6 months. One of the animals was 39 months old, and presented a weight of 3.2 kg, while the other was 29 months old and presented a weight of 11 kg. Before the beginning of the study, the animal’s clinical evaluation was performed, being confirmed that neither presented periodontal disease or other concomitant diseases.

### Sequencing data

A total of 8,453,453 reads were obtained from the 12 samples collected, with a mean value of 704,454.4 ± 118,960.7. For alfa rarefaction analysis a total of 15,37,563 reads remained for diversity analysis (Supplemental File 1).

### Diversity analysis

Analysis of the diversity of samples collected in different timepoints was performed with Shannon index. All samples presented a Shannon index between 7 and 8, corresponding to a high diversity. The Shannon index was higher in the T1 samples, followed by the ones from T3 and T2.

Statistically significant differences were observed in the Kruskal–Wallis pairwise group evaluation, between microbial diversity present in T1 and T2 samples (*p*-value = 0.043) and between T2 and T3 samples (*p*-value = 0.043).

### Taxonomic diversity

Taxonomy classification assigned using the Greenegene99 database allowed to obtain a total of 18 phylum, 24 classes, 32 orders, 49 families, 58 genus and 26 species in the samples under study. The richness of each individual sample is presented in Table 1. In the samples collected before the applications of the nisin-biogel (T1), representing the commensal oral microbiome, ten phyla had relative sequence abundances higher than 1%: Bacteroidetes (40.97%), Proteobacteria (22.36%), Fusobacteria (7.78%), Firmicutes (7.10%), Spirochaetes (7.02%), SRI (4.89%), Tenericutes (3.43%), GN02 (2.21%), TM7 (1.84%) and Actinobacteria (1.56%).

**Table 1:** Taxonomic richness of samples: distribution of the number of each taxonomic level identified by sample.

Sample ID	Taxonomic level					
	Phylum	Class	Order	Family	Genus	Species
T1_Va	17	24	31	46	49	19
T1_Vb	18	23	30	46	48	17
T1_Ca	14	21	27	44	50	20
T1_Cb	14	21	28	46	50	18
T2_Va	17	23	30	45	48	18
T2_Vb	18	23	29	44	48	16
T2_Ca	14	21	28	46	51	19
T2_Cb	14	21	28	44	51	19
T3_Va	18	24	30	48	50	16
T3_Vb	18	23	29	46	49	17
T3_Ca	14	21	28	45	51	19
T3_Cb	14	21	26	45	50	18
Total	18	24	32	49	58	26

Note:

T1—samples collected before nisin-biogel applications; T2—samples collected after one application of the nisin-biogel; T3—samples collected after three applications of the nisin-biogel; ID—identification. Va—Animal V, sample 1; Vb—Animal V, sample 2; Ca—Animal C, sample 1; Cb— Animal C, sample 2.

**Figure 1:**
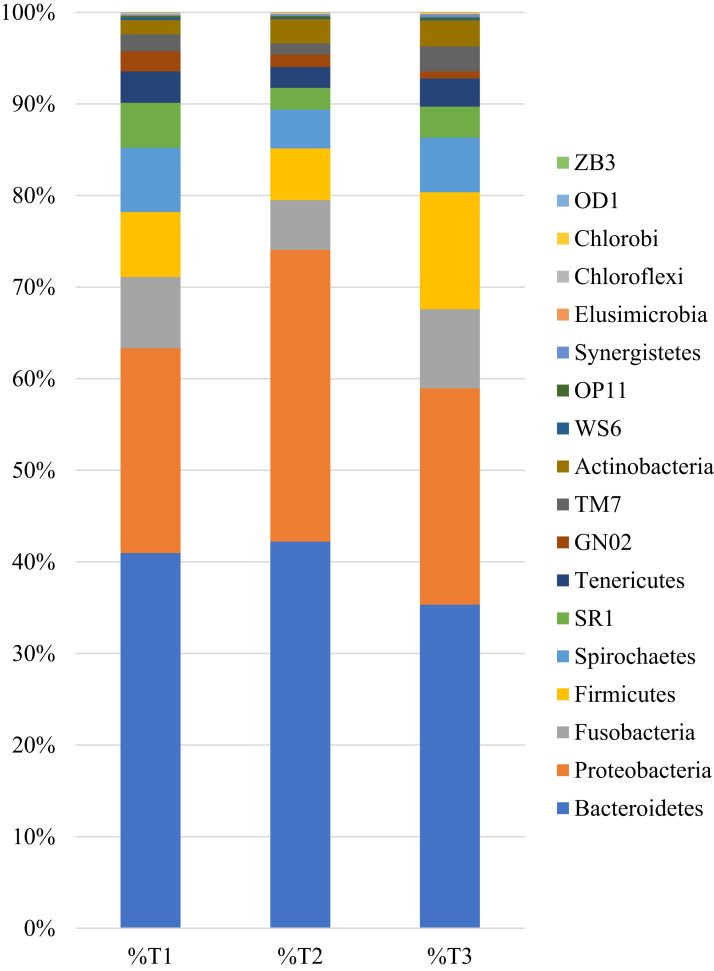
Relative distribution of sequences obtained from the 12 samples by Phylum level, in each timepoint. T1—samples collected before nisin-biogel application; T2—samples collected after one application of the nisin-biogel; T3—samples collected after three applications of the nisin-biogel.

In the samples collected after one (T2) and three (T3) applications of the compound, the same ten phyla were the most abundant. The samples taken at timepoint T2 presented 42.21% of Bacteroidetes, 31.87% of Proteobacteria, 5.64% of Firmicutes, 5.44% of Fusobacteria, 4.21% of Spirochaetes, 2.63% of Actinobacteria, 2.39 % of SRI, 2.27% of Tenericutes, 1.37% of GN02 and 1.24% TM7. The samples from timepoint T3 presented 35.32% of Bacteroidetes, 23.61% of Proteobacteria, 12.79% of Firmicutes, 8.65% of Fusobacteria, 5.98% of Spirochaetes, 3.36% of SRI, 3.05% of Tenericutes, 2.82% of Actinobacteria, 2.71% TM7 and 0.80% of GN02 (Fig. 1).

Detailed analysis was performed on the species level, and the most prevalent bacterial species were investigated to understand their potential relation with PD in dogs.

### Species abundance

**Figure 2:**
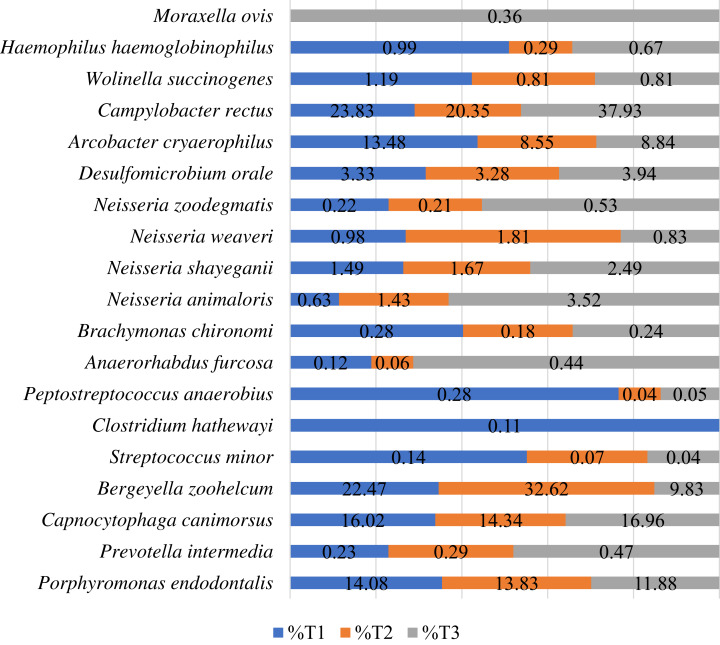
Relative distribution of the sequences at the species level by timepoint. Only species corresponding to more than 0.1% of the sequences are presented. T1—samples collected before nisin-biogel application (number of total reads = 67,478); T2—samples collected after one application of the nisin-biogel (number of total reads = 95,633); T3—samples collected after three applications of the nisin-biogel (number of total reads = 68,647).

A total of 26 species were identified in the 12 samples evaluated. Species with a total relative abundance higher than 0.1% are presented in Fig. 2. Classification at the species level was only possible for 2,31,758 reads, from a total of 15,37,563 reads.

The commensal oral microbiome (T1) showed a high prevalence of *Campylobacter rectus*, *Bergeyella zoohelcum*, *Capnocytophaga canimorsus*, *Porphyromonas endodontalis* and *Arcobacter cryaerophylus*. After one application of nisin-biogel (T2) the most prevalent species identified were the same as in T1. Furthermore, most species showed a prevalence reduction after one application of nisin-biogel, except for *B. zoohelcum*, *N. weaveri*., *N. shayeganii*, *N. animaloris* and *Prevotella intermedia*.

Comparing the species abundance in samples collected at timepoint T3 with the ones from T1, it was possible to observe a reduction of prevalence in the following species *Porphyromonas endodontalis*, *Bergeyella zoohelcum*, *N. weaveri*, *Peptostreptococcus anaerobius*, *Streptococcus minor*, *Arcobacter cryaerophylus*, *Haemophilus haemoglobinophilus*, *Wollinela succinogenes* and *Brachymonas chironomi* (Fig. 2).

## Discussion

The canine oral microbiome is a complex environment, which composition can be influenced by external or individual factors, such as alimentary regimens, oral hygiene procedures, individual genetic features or oral health status (Riggio et al., 2011; Wallis et al., 2015; Davis, 2016; Flancman, Singh & Weese, 2018; Ruparell et al., 2020; Wu et al., 2020). Research in this area can help to understand the implications that some microorganisms can have in disease development or the influence of antimicrobial compounds in the oral microbiome.

In this study, we aimed to understand the impact of the topical application of a nisin-biogel on the tooth surface composite microbiome of dogs. Diversity analysis showed that the commensal oral microbiome presented the higher Shannon index, followed by the samples collected after three nisin-biogel applications (T3) and the samples collected after one application of the nisin-biogel (T2). It was expected that oral samples without any disturbance presented a higher richness and evenness when compared with samples submitted to antimicrobials. Nisin-biogel is a compound that combines the antimicrobial activity of nisin and the delivery capacity of guar gum (Cunha et al., 2020). Its use in canine dental topical applications is being proposed by our team, after a series of promising results regarding its antimicrobial ability, stability and safety (Cunha et al., 2018; Cunha et al., 2020). It was interesting to verify that after only one application of the nisin-biogel, a statistically significant reduction in bacterial diversity in the oral cavity of the dogs under study was observed. However, after three applications of the nisin-biogel (one week of applications), an increased diversity was observed, showing a recovery of the microbial diversity. This result is very promising, allowing us to hypothesize that a continuous application of the nisin-biogel will have minimal effects on the oral commensal microbiome.

Taxonomic richness evaluation allowed to identify a total of 18 Phylum. The samples collected prior to the applications revealed a major representation of Bacteroidetes (40.97%), Proteobacteria (22.36%), Fusobacteria (7.78%), Firmicutes (7.10%) and Spirochaetes (7.02%), in accordance with other studies on the canine oral microbiome (Dewhirst et al., 2012; Davis et al., 2013; Davis, 2016; McDonald et al., 2016). Flancman, Singh & Weese (2018) described Proteobacteria (58.3%) and Firmicutes (19.1%) as the most represented Phylum in their oral microbiome evaluation (Flancman, Singh & Weese, 2018). Also, in 2020, Ruparell stated that Proteobacteria (32.8%), Firmicutes (27.5%), Bacteroidetes (17.5%), Actinobacteria (4.5%) and Fusobacteria (2%) were the most represented phylum in the canine oral microbiome (Ruparell et al., 2020). In our study, after the application of the nisin-biogel some differences were observed in the relative abundance of these phyla, but no changes in their general representability. These results are in accordance with Wu and collaborators (2020), who studied the influence of nisin food supplementation in the oral microbiome of rats, observing that nisin can lightly affect the diversity and composition of the rat oral microbiota (Wu et al., 2020).

Since nisin-biogel has been evaluated as a new compound for PD control in dogs, a detailed analysis was performed on the species level and the most prevalent bacterial species were investigated to understand their potential relation with PD in dogs.

Considering the samples species richness, only 2,31,748 reads were allocated to one of the 26 species identified. The ones with more representability in all timepoints were *Campylobacter rectus*, *Bergeyella zoohelcum*, *Capnocytophaga canimorsus*, *Porphyromonas endodontalis* and *Arcobacter cryaerophilus*.

*Campylobacter rectus* is a microaerophilic, gram-negative, rod bacteria, that belongs to the normal human oral subgingival microbiota (Lee et al., 2016). In humans, *C. rectus* is related to the onset of periodontal disease, but also with various gastrointestinal diseases and extraintestinal infections (Kato et al., 2011; Lee et al., 2016). *C. rectus* was already detected in the oral cavity of dogs using molecular methods and it has been suggested a possible link with periodontitis, but its clinical significance in those animals is still uncertain (Bojanić et al., 2019; Kačírová et al., 2019). In this study, a reduction of *C. rectus* was observed after one application of the nisin-biogel, but a recovery and increase of this species was observed after one week of applications.

Other bacteria identified was *Bergeyella zoohelcum*, an aerobic, gram-negative, rod bacteria, belonging to the oral microbiota of dogs, cats and other mammals (Muramatsu et al., 2019). Several reports have identified *B. zoohelcum* in the supragingival plaque of dogs with and without PD, with prevalences ranging from 13.3% to 90% (Holcombe et al., 2014; Muramatsu et al., 2019; Ruparell et al., 2020). In fact, Holcombe et al. (2014) described it as the taxa with the highest relative abundance in early plaque biofilms *in vivo*, which may act as an early colonizer in the establishment of canine PD. In this study, *B. zoohelcum* presented a high decrease, from 22.47% in the samples representing the commensal oral microbiome (T1), to 9.83% in the samples collected after three applications of the nisin-biogel. As this species may influence the initial dental plaque formation, its reduction can help to prevent PD development in dogs. However, other early colonizers of the canine dental plaque should be considered, as *Neisseria*, *Moraxella* or *Corynebacterium* species (Holcombe et al., 2014; Davis, 2016). From all the four species of *Neisseria* and one of *Moraxella* identified in our study, only *N. weaverii* showed a prevalence reduction after one week of nisin-biogel application (T3). The reduction of some considered early colonizers may be an indicator of the potential action of nisin-biogel in the inhibition of dental plaque formation. In addition, *B. zoohelcum* is a zoonotic pathogen, causing local infection or even bacteraemia in humans after dog’s bite, being interesting its reduction after nisin-biogel administration (Chen et al., 2017).

Showing a relative abundance of 16.02% in the samples collected before application of the nisin-biogel, *Capnocytophaga canimorsus* is a gram-negative capnophilic rod, that belongs to the commensal oral microbiota of dogs and cats (Zajkowska et al., 2016). Despite *C. canimorsus* does not seem to cause disease in dogs, other *Capnocytophaga* species have been linked to PD in humans (Zajkowska et al., 2016). Also, it can cause infections in humans after dog’s bite and scratch, or through close contact with those animals (Umeda et al., 2014; Zajkowska et al., 2016). Besides its reduction after one nisin-biogel application (T2), a recovery of its abundance was observed at T3.

*Porphyromonas* is a frequent genus in canine oral microbiome. *P. cangingivalis* is described as the most abundant species in the dental plaque, being identified in disease or non-disease periodontium, due to its metabolic flexibility (Davis et al., 2013; O’Flynn et al., 2015; Kačírová et al., 2019; Ruparell et al., 2020). Also, *P. gulae*, present in periodontitis, is an important pathogen in PD progression (Kačírová et al., 2019). In this study, *P. endodontalis* was the only *Porphyromonas* species identified, being usually found in endodontic infections in humans, but rarely seen in dogs (Tavana, 2009). However, in 2015, O’Flynn and its colleagues performed a comparative genetic analysis of the genus *Porphyromonas* and described a high similarity between *P. endodontalis* and *P. gingivicanis*, which may explain its high abundance in the samples analyzed (O’Flynn et al., 2015). Despite that, a progressive reduction of this microorganism was observed after the nisin-biogel application. A reduction of other *Porphyromonas* species, such as *P. cangingivalis* or *P. gulae*, would also support nisin-biogel application as a PD control measure.

Another species with high prevalence in the commensal samples (13.48%) was *Arcobacter cryaerophilus*. A reduction of its prevalence was observed after nisin-biogel application (T2 and T3). *A. cryaerophilus* was already found in the oral cavity of dogs, but there is no description of its relationship with oral diseases (Houf et al., 2008). In fact, this microaerophilic gram-negative bacterium is usually found on foods of animal origin or wastewater, and it is associated with enteritis and septicemia in humans (Houf et al., 2008; Kristensen et al., 2020).

Finally, it is important to refer that a reduction in *Peptostreptococcus anaerobius* after nisin-biogel administration was observed. This bacterium is a gram-positive, anaerobic coccus, potentially present in mild (PD stage 2) to severe cases of periodontitis (PD stage 3 and 4) in dogs (Lawson et al., 2012; Davis et al., 2013; Polkowska, Sobczyńska-Rak & Gołyńska, 2014).

In conclusion, this pilot study showed that nisin-biogel application have a slight influence in the canine oral microbiome. In fact, it was possible to observe that the diversity of the commensal oral microbiota suffers a statistically significant reduction after one nisin-biogel application, however after three nisin-biogel applications the diversity seems to recovery. Also, there was a reduction in some bacteria that can be related to distinct PD stages, being these reductions a positive suggestion that the nisin-biogel may be a potential compound to be used as a PD control measure in dogs. Nevertheless, the oral cavity is a complex and diverse environment, being the oral microbiome only a piece in PD development. In fact, host immune system and local oral environment are essential factors in PD establishment in dogs (Niemiec, 2008; Teles et al., 2013; Wu et al., 2020). The immuno-inflammatory response is a major mechanism of periodontal destruction and PD progression (Teles et al., 2013; Stepaniuk, 2019), so the evaluation of nisin-biogel influence on this system would be important in upcoming studies.

In the future, it will be essential to evaluate the nisin-biogel activity under a long-term administration period and in a controlled *in vivo* clinical trial, to evaluate periodontal indices evolution, such as gingival index, dental plaque accumulation, furcation exposure, clinical attachment level, probing pocket depth, furcation exposure or even tooth mobility.

## Conclusions

PD is a common inflammatory disease in dogs, resulting from a polymicrobial biofilm in the teeth surface and a host immune-inflammatory response. The nisin-biogel under evaluation has been described as a potential compound to be used in the control of canine PD. Being an antimicrobial compound, the nisin-biogel application on the dental surface may affect not only the bacteria responsible for PD onset but also the remaining oral commensal bacteria. This pilot study allowed to observe that nisin-biogel may promote some changes in the oral microbiome of dogs. After one application of the nisin-biogel, a statistically significant reduction in microbial diversity was observed. Nevertheless, after a total of three applications of the nisin-biogel, a recovery of the microbial diversity was detected. In association with other studies previously performed to access nisin-biogel as a potential compound to be used in the management of canine PD, this pilot study helped to evaluate its effect on the commensal oral microbiome in dogs, with results to be considered in the future design of a larger controlled clinical long-term trial.

## Supplemental Information

10.7717/peerj.11626/supp-1 Supplemental Information 1 Distribution of demultiplexed reads and reads used in taxa classification by sample and by timepoint. T1—samples collected before nisin-biogel application; T2—after one application of the nisin-biogel; T3—after three applications of the nisin-biogel. SD—standard deviation; ID—identification.

10.7717/peerj.11626/supp-2 Supplemental Information 2 Raw material regarding Filum and Species identifcation. Description of the number of reads obtained by sample and taxonomic level. Filum and Species were the taxonomic levels evaluated.

## Additional Information and Declarations

### Data Availability

The following information was supplied regarding data availability: The distribution of demultiplexed reads and reads used in taxa classification by sample and by timepoint, and the datasets used, are available in the Supplemental Files. The original sequence datasets are available at NCBI SRA: PRJNA703322.
