# Supplementary material for: Influence of the dental topical application of a nisin-biogel in the oral microbiome of dogs: a pilot study
Source: PeerJ. 2021 Jul 14;9:e11626. doi: 10.7717/peerj.11626 (PMC8286056; doi:10.7717/peerj.11626)
Supplement: Supplemental Information 1 — T1—samples collected before nisin-biogel application; T2—after one application of the nisin-biogel; T3—after three applications of the nisin-biogel. SD—standard deviation; ID—identification. [file peerj-09-11626-s001.docx]

**Supplementary file 1:** Distribution of demultiplexed reads and reads used in taxa classification by sample and by timepoint.

| Sample ID | Demultiplexed reads | Taxa reads |
| --- | --- | --- |
| T1_Va | 824306 | 149254 |
| T1_Vb | 577888 | 104747 |
| T1_Ca | 647315 | 109238 |
| T1_Cb | 684246 | 125579 |
| T2_Va | 592004 | 105268 |
| T2_Vb | 526664 | 111542 |
| T2_Ca | 591008 | 102640 |
| T2_Cb | 778140 | 154520 |
| T3_Va | 870388 | 152532 |
| T3_Vb | 773726 | 143650 |
| T3_Ca | 867128 | 151139 |
| T3_Cb | 720640 | 127454 |
| Total | 8453453 | 1537563 |
| Mean±SD | 704454.4±118960.7 | 128130.3±21033.76 |
| T1 | 2733755 | 488818 |
| T2 | 2487816 | 473970 |
| T3 | 3231882 | 574775 |

T1 – samples collected before nisin-biogel application; T2 – after one application of the nisin-biogel; T3- after three applications of the nisin-biogel. SD – standard deviation; ID – identification.
